# Multi-Objective Evolutionary Rule-Based Classification with Categorical Data

**DOI:** 10.3390/e20090684

**Published:** 2018-09-07

**Authors:** Fernando Jiménez, Carlos Martínez, Luis Miralles-Pechuán, Gracia Sánchez, Guido Sciavicco

**Affiliations:** 1 Department of Information and Communication Engineering, University of Murcia, 30071 Murcia, Spain; 2 Centre for Applied Data Analytics Research (CeADAR), University College Dublin, D04 Dublin 4, Ireland; 3 Department of Mathematics and Computer Science, University of Ferrara, 44121 Ferrara, Italy

**Keywords:** multi-objective evolutionary algorithms, rule-based classifiers, interpretable machine learning, categorical data

## Abstract

The ease of interpretation of a classification model is essential for the task of validating it. Sometimes it is required to clearly explain the classification process of a model’s predictions. Models which are inherently easier to interpret can be effortlessly related to the context of the problem, and their predictions can be, if necessary, ethically and legally evaluated. In this paper, we propose a novel method to generate rule-based classifiers from categorical data that can be readily interpreted. Classifiers are generated using a multi-objective optimization approach focusing on two main objectives: maximizing the performance of the learned classifier and minimizing its number of rules. The multi-objective evolutionary algorithms *ENORA* and *NSGA-II* have been adapted to optimize the performance of the classifier based on three different machine learning metrics: accuracy, area under the *ROC* curve, and root mean square error. We have extensively compared the generated classifiers using our proposed method with classifiers generated using classical methods such as *PART*, *JRip*, *OneR* and *ZeroR*. The experiments have been conducted in full training mode, in 10-fold cross-validation mode, and in train/test splitting mode. To make results reproducible, we have used the well-known and publicly available datasets *Breast Cancer*, *Monk’s Problem 2*, *Tic-Tac-Toe-Endgame*, *Car*, *kr-vs-kp* and *Nursery*. After performing an exhaustive statistical test on our results, we conclude that the proposed method is able to generate highly accurate and easy to interpret classification models.

## 1. Introduction

*Supervised Learning* is the branch of *Machine Learning* (*ML*) [1] focused on modeling the behavior of systems that can be found in the environment. Supervised models are created from a set of past records, each one of which, usually, consists of an input vector labeled with an output. A supervised model is an algorithm that simulates the function that maps inputs with outputs [2]. The best models are those that predict the output of new inputs in the most accurate way. Thanks to modern computing capabilities, and to the digitization of ever-increasing quantities of data, nowadays, supervised learning techniques play a leading role in many applications. The first classification systems date back to the 1990s; in those days, researchers were focused on both precision and interpretability, and the systems to be modeled were relatively simple. Years later, when it became necessary to model more difficult behaviors, the researchers focused on developing more and more precise models, leaving aside the interpretability. *Artificial Neural Networks* (*ANN*) [3], and, more recently, *Deep Learning Neural Networks* (*DLNN*) [4], as well as *Support Vector Machines* (*SVM*) [5], and *Instance-based Learning* (*IBL*) [6] are archetypical examples of this approach. A *DLNN*, for example, is a large mesh of ordered nodes arranged in a hierarchical manner and composed of a huge number of variables. *DLNN*s are capable of modeling very complex behaviors, but it is extremely difficult to understand the logic behind their predictions, and similar considerations can be drawn for *SVN*s and *IBL*s, although the underlying principles are different. These models are known as *black-box* methods. While there are applications in which knowing the ratio behind a prediction is not necessarily relevant, (e.g., predicting a currency’s future value, whether or not a user clicks on an advert or the amount of rain in a certain area), there are other situations where the interpretability of a model plays a key role.

The *interpretability* of classification systems refers to the ability they have to explain their behavior in a way that is easily understandable by a user [7]. In other words, a model is considered interpretable when a human is able to understand the logic behind its prediction. In this way, Interpretable classification models allow external validation by an expert. Additionally, there are certain disciplines such as medicine, where it is essential to provide information about decision making for ethical and human reasons. Likewise, when a public institution asks an authority for permission to investigate an alleged offender, or when the CEO of a certain company wants to take a difficult decision which can seriously change the direction of the company, some kind of explanations to justify these decisions may be required. In these situations, using transparent (also called grey-box) models is recommended. While there is a general consensus on how the performance of a classification system is measured (popular metrics include *accuracy*, *area under the ROC curve*, and *root mean square error*), there is no universally accepted metric to measure the interpretability of the models. Nor is there an ideal balance between the interpretability and performance of classification systems but this depends on the specific application domain. However, the rule of thumb says that the simpler a classification system is, the easier it is to interpret. *Rule-based Classifiers* (*RBC*) [8,9] are among the most popular interpretable models, and some authors define the degree of interpretability of an *RBC* as the number of its rules or as the number of axioms that the rules have. These metrics tend to reward models with fewer rules as simple as possible [10,11]. In general, *RBC*s are classification learning systems that achieve a high level of interpretability because they are based on a human-like logic. Rules follow a very simple schema:

*IF (Condition 1) and (Condition 2) and … (Condition N) THEN (Statement)*

and the fewer rules the models have and the fewer conditions and attributes the rules have, the easier it will be for a human to understand the logic behind each classification. In fact, *RBC*s are so natural in some applications that they are used to interpret other classification models such as *Decision Trees* (*DT*) [12]. *RBC*s constitute the basis of more complex classification systems based on fuzzy logic [13] such as *LogitBoost* or *AdaBoost* [14].

Our approach investigates the conflict between accuracy and interpretability as a *multi-objective optimization problem*. We define a solution as a set of rules (that is, a classifier), and establish two objectives to be maximized: interpretability and accuracy. We decided to solve this problem by applying *multi-objective evolutionary algorithms* (*MOEA*) [15,16] as meta-heuristics, and, in particular, two known algorithms: *NSGA-II* [15] and *ENORA* [17]. They are both state-of-the-art evolutionary algorithms which have been applied, and compared, on several occasions [18,19,20]. *NSGA-II* is very well-known and has the advantage of being available in many implementations, while *ENORA* generally has a higher performance. In the current literature, *MOEA*s are mainly used for learning *RBC*s based on fuzzy logic [18,21,22,23,24,25,26]. However, *Fuzzy RBC*s are designed for numerical data, from which fuzzy sets are constructed and represented by linguistic labels. In this paper, on the contrary, we are interested in *RBC*s for categorical data, for which a novel approach is necessary.

This paper is organized as follows. In Section 2, we introduce multi-objective constrained optimization, the evolutionary algorithms *ENORA* and *NSGA-II*, and the well-known rule-based classifier learning systems *PART*, *JRip*, *OneR* and *ZeroR*. In Section 3, we describe the structure of an *RBC* for categorical data, and we propose the use of multi-objective optimization for the task of learning a classifier. In Section 4, we show the result of our experiments, performed on the well-known publicly accessible datasets *Breast Cancer*, *Monk’s Problem 2*, *Tic-Tac-Toe-Endgame*, *Car*, *kr-vs-kp* and *Nursery*. The experiments allow a comparison among the performance of the classifiers learned by our technique against those of classifiers learned by *PART*, *JRip*, *OneR* and *ZeroR*, as well as a comparison between *ENORA* and *NSGA-II* for the purposes of this task. In Section 5, the results are analyzed and discussed, before concluding in Section 6. Appendix A and Appendix B show the tables of the statistical tests results. Appendix C shows the symbols and the nomenclature used in the paper.

## 2. Background

### 2.1. Multi-Objective Constrained Optimization

The term *optimization* [27] refers to the selection of the best element, with regard to some criteria, from a set of alternative elements. *Mathematical programming* [28] deals with the theory, algorithms, methods and techniques to represent and solve optimization problems. In this paper, we are interested in a class of mathematical programming problems called *multi-objective constrained optimization problems* [29], which can be formally defined, for *l* objectives and *m* constraints, as follows:

(1) $$\begin{array}{ccc} Min./Max. & f_{i}\left(x\right), & i=1,\;\ldots ,\;l\\ subject\;to & g_{j}\left(x\right)\leq 0, & j=1,\;\ldots ,\;m \end{array}$$

where $f_{i}\left(x\right)$ (usually called *objectives*) and $g_{j}\left(x\right)$ are arbitrary functions. Optimization problems can be naturally separated into two categories: those with discrete variables, which we call *combinatorial*, and those with continuous variables. In combinatorial problems, we are looking for objects from a finite, or countably infinite, set X, where objects are typically integers, sets, permutations, or graphs. In problems with continuous variables, instead, we look for real parameters belonging to some continuous domain. In Equation (1), $x=\left\{x_{1},\;x_{2},\;\ldots ,\;x_{w}\right\}\in X^{w}$ represents the set of decision variables, where X is the domain for each variable $x_{k}$, k=1,…,w.

Now, let $F=\{x\in X^{w}\;|\;g_{j}\left(x\right)\leq 0,\;j=1,\;\ldots ,\;m\}$ be the set of all feasible solutions to Equation (1). We want to find a subset of solutions S⊆F called *non-dominated set* (or *Pareto optimal set*). A solution x∈F is *non-dominated* if there is no other solution $x^{\prime }\in F$ that dominates x, and a solution $x^{\prime }$ *dominates* x if and only if there exists *i* (1≤i≤l) such that $f_{i}\left(x^{\prime }\right)$ improves $f_{i}\left(x\right)$, and for every *i* (1≤i≤l), $f_{i}\left(x\right)$ does not improve $f_{i}\left(x^{\prime }\right)$. In other words, $x^{\prime }$ *dominates* x if and only if $x^{\prime }$ is better than x for at least one objective, and not worse than x for any other objective. The set S of non dominated solutions of Equation (1) can be formally defined as:

$$S=\left\{x\in F\;|∄\;x^{\prime }\left(x^{\prime }\in F\wedge D\left(x^{\prime },x\right)\right)\right\}$$

where:

$$D\left(x^{\prime },x\right)=\exists i\left(1\leq i\leq l,f_{i}\left(x^{\prime }\right)<f_{i}\left(x\right)\right)\wedge \forall i\left(1\leq i\leq l,f_{i}\left(x^{\prime }\right)\leq f_{i}\left(x\right)\right).$$

Once the set of optimal solutions is available, the most satisfactory one can be chosen by applying a preference criterion. When all the functions $f_{i}$ are linear, then the problem is a *linear programming problem* [30], which is the classical mathematical programming problem and for which extremely efficient algorithms to obtain the optimal solution exist (e.g., the *simplex method* [31]). When any of the functions $f_{i}$ is non-linear then we have a *non-linear programming problem* [32]. A non-linear programming problem in which the objectives are arbitrary functions is, in general, intractable. In principle, any search algorithm can be used to solve combinatorial optimization problems, although it is not guaranteed that they will find an optimal solution. *Metaheuristics* methods such as *evolutionary algorithms* [33] are typically used to find approximate solutions for complex multi-objective optimization problems, including feature selection and fuzzy classification.

### 2.2. The Multi-Objective Evolutionary Algorithms ENORA and NSGA-II

The *MOEA ENORA* [17] and *NSGA-II* [15] use a $\left(\mu +\lambda \right)$ strategy (Algorithm 1) with μ=λ=popsize, where μ corresponds to the number of parents and λ refers to the number of children (popsize is the population size), with *binary tournament selection* (Algorithm 2) and a rank function based on Pareto fronts and *crowding* (Algorithms 3 and 4). The difference between *NSGA-II* and *ENORA* is how the calculation of the ranking of the individuals in the population is performed. In *ENORA*, each individual belongs to a slot (as established in [34]) of the objective search space, and the rank of an individual in a population is the non-domination level of the individual in its slot. On the other hand, in *NSGA-II*, the rank of an individual in a population is the non-domination level of the individual in the whole population. Both *ENORA* and *NSGA-II* MOEAs use the same non-dominated sorting algorithm, the *fast non-dominated sorting* [35]. It compares each solution with the rest of the solutions and stores the results so as to avoid duplicate comparisons between every pair of solutions. For a problem with *l* objectives and a population with *N* solutions, this method needs to conduct l·N·(N−1) objective comparisons, which means that it has a time complexity of $O(l\cdot N^{2})$ [36]. However, *ENORA* distributes the population in *N* slots (in the best case), therefore, the time complexity of *ENORA* is $O(l\cdot N^{2})$ in the worst case and O(l·N) in the best case.

**Algorithm 1**$\left(\mu +\lambda \right)$ strategy for multi-objective optimization.**Require:**T>1 {Number of generations} **Require:**
N>1 {Number of individuals in the population}
 1:Initialize *P* with *N* individuals 2:Evaluate all individuals of *P* 3:t←0 4:**while**t<T**do** 5: Q←∅ 6: i←0 7: **while** i<N **do** 8:  *Parent1*← Binary tournament selection from *P* 9:  *Parent2*← Binary tournament selection from *P*10:  *Child1*, *Child2*←*Crossover*(*Parent1*, *Parent2*)11:  *Offspring1*←*Mutation*(*Child1*)12:  *Offspring2*←*Mutation*(*Child2*)13:  Evaluate *Offspring1*14:  Evaluate *Offspring2*15:  $Q\leftarrow Q⋃\left\{\mathrm{Offspring}1,\mathrm{Offspring}2\right\}$16:  i←i+217: **end while**18: R←P⋃Q19: P←*N* best individuals from *R* according to the *rank-crowding* function in population *R*20: t←t+121:**end while**22:**return** Non-dominated individuals from *P*

**Algorithm 2** Binary tournament selection.**Require:***P* {Population}
 1:I← Random selection from *P* 2:J← Random selection from *P* 3:**if***I* is better than *J* according to the *rank-crowding* function in population *P* **then** 4: **return** *I* 5:**else** 6: **return** *J* 7:**end if**


**Algorithm 3** Rank-crowding function.**Require:***P* {Population} **Require:**
I,J {Individuals to compare}
 1:**if**$rank\left(P,I\right)<rank\left(P,J\right)$**then** 2: **return** True 3:**end if** 4:**if**rank(P,J)<rank(P,I)**then** 5: **return** False 6:**end if** 7:**return**$Crowding\_distance\left(P,I\right)>Crowding\_distance\left(P,J\right)$


**Figure 1:**
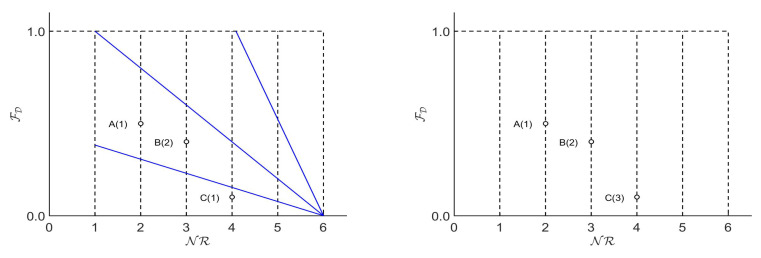
Rank assignment of individuals with *ENORA* vs. *NSGA-II*.

The main reason *ENORA* and *NSGA-II* behave differently is as follows. *NSGA-II* never selects the individual dominated by the other in the binary tournament, while, in *ENORA*, the individual dominated by the other may be the winner of the tournament. Figure 1 shows this behavior graphically. For example, if individuals *B* and *C* are selected for a binary tournament with *NSGA-II*, individual *B* beats *C* because *B* dominates *C*. Conversely, individual *C* beats *B* with *ENORA* because individual *C* has a better rank in his slot than individual *B*. In this way, *ENORA* allows the individuals in each slot to evolve towards the Pareto front encouraging diversity. Even though in *ENORA* the individuals of each slot may not be the best of the total individuals, this approach generates a better hypervolume than that of *NSGA-II* throughout the evolution process.

*ENORA* is our *MOEA*, on which we are intensively working over the last decade. We have applied *ENORA* to constrained real-parameter optimization [17], fuzzy optimization [37], fuzzy classification [18], feature selection for classification [19] and feature selection for regression [34]. In this paper, we apply it to rule-based classification. *NSGA-II* algorithm was designed by Deb et al. and has been proved to be a very powerful and fast algorithm in multi-objective optimization contexts of all kinds. Most researchers in multi-objective evolutionary computation use *NSGA-II* as a baseline to compare the performance of their own algorithms. Although *NSGA-II* was developed in 2002 and remains a state-of-the-art algorithm, it is still a challenge to improve on it. There is a recently updated improved version for *many-objective optimization* problems called *NSGA-III* [38].

**Algorithm 4** Crowding_distance function.**Require:***P* {Population} **Require:**
*P* {Population} **Require:**
*I* {Individual} **Require:**
*l* {Number of objectives}
 1:**for**j=1 to *l* **do** 2: $f_{j}^{max}\leftarrow \max_{I\in P}\left\{f_{j}^{I}\right\}$ 3: $f_{j}^{min}\leftarrow \min_{I\in P}\left\{f_{j}^{I}\right\}$ 4: $f_{j}^{sup_{j}^{I}}\leftarrow$ value of the *j*th objective for the individual higher adjacent in the *j*th objective to the individual *I* 5: $f_{j}^{inf_{j}^{I}}\leftarrow$ value of the *j*th objective for the individual lower adjacent in the *j*th objective to the individual *I* 6:**end for** 7:**for**j=1 to *l* **do** 8: **if** $f_{j}^{I}=f_{j}^{max}$ or $f_{j}^{I}=f_{j}^{min}$ **then** 9:  **return** ∞10: **end if**11:**end for**12:CD←0.013:**for**j=1 to *l* **do**14: $CD\leftarrow CD+\frac{f_{j}^{sup_{j}^{I}}-f_{j}^{inf_{j}^{I}}}{f_{j}^{max}-f_{j}^{min}}$15:**end for**16:**return**CD


### 2.3. PART

*PART* (*Partial DT Method* [39]) is a widely used rule learning algorithm that was developed at the University of Waikato in New Zealand [40]. Experiments show that it is a very efficient algorithm in terms of both computational performance and results. *PART* combines the divide-and-conquer strategy typical of decision tree learning with the separate-and-conquer strategy [41] typical of rule learning, as follows. A decision tree is first constructed (using *C4.5* algorithm [42]), and the leaf with the highest coverage is converted into a rule. Then, the set of instances that are covered by that rule are discarded, and the process starts over. The result is an ordered set of rules, completed by a *default* rule that applies to instances that do not meet any previous rule.

### 2.4. JRip

*JRip* is a fast and optimized implementation in *Weka* of the famous *RIPPER* (*Repeated Incremental Pruning to Produce Error Reduction*) algorithm [43]. *RIPPER* was proposed in [44] as a more efficient version of the incrementally reduced error pruning (*IREP*) rule learner developed in [45]. *IREP* and *RIPPER* work in a similar manner. They begin with a default rule and, using a training dataset, attempt to learn rules that predict exceptions to the default. Each rule learned is a conjunction of propositional literals. Each literal corresponds to a split of the data based on the value of a single feature. This family of algorithms, similar to decision trees, has the advantage of being easy to interpret, and experiments show that *JRip* is particularly efficient in large datasets. *RIPPER* and *IREP* use a strategy based on the separate-and-conquer method to generate an ordered set of rules that are extracted directly from the dataset. The classes are examined one by one, prioritizing those that have more elements. These algorithms are based on four basic steps (growing, pruning, optimizing and selecting) applied repetitively to each class until a stopping condition is met [44]. These steps can be summarized as follows. In the growing phase, rules are created taking into account an increasing number of predictors until the stopping criterion is satisfied (in the *Weka* implementation, the procedure selects the condition with the highest information gain). In the pruning phase redundancy is eliminated and long rules are reduced. In the optimization phase, the rules generated in the previous steps are improved (if possible) by adding new attributes or by adding new rules. Finally, in the selection phase, the best rules are selected and the others discarded.

### 2.5. OneR

*OneR* (*One Rule*) is a very simple, while reasonably accurate, classifier based on a frequency table. First, *OneR* generates a set of rules for each attribute of the dataset, and, then, it selects only one rule from that set—the one with the lowest error rate [46]. The set of rules is created using a frequency table constructed for each predictor of the class, and numerical classes are converted into categorical values.

### 2.6. ZeroR

Finally, *ZeroR* (*Zero Rules* [40]) is a classifier learner that does not create any rules and uses no attributes. *ZeroR* simply creates the class classification table by selecting the most frequent value. Such a classifier is obviously the simplest possible one, and its capabilities are limited to the prediction of the majority class. In the literature, it is not used for practical classifications tasks, but as a generic reference to measure the performance of other classifiers.

## 3. Multi-Objective Optimization for Categorical Rule-Based Classification

In this section, we propose a general schema for an *RBC* specifically designed for categorical data. Then, we propose and describe a multi-objective optimization solution to obtain optimal categorical *RBC*s.

### 3.1. Rule-Based Classification for Categorical Data

Let Γ be a classifier composed by *M* rules, where each rule $R_{i}^{\Gamma }$, i=1,…,M, has the following structure:

(2) $$\begin{array}{ccccccccc} R_{i}^{\Gamma }: & IF & x_{1}=b_{i1}^{\Gamma } & AND & ,\;\ldots , & AND & x_{p}=b_{ip}^{\Gamma } & THEN & y=c_{i}^{\Gamma } \end{array}$$

where for j=1,…,p the attribute $b_{ij}^{\Gamma }$ (called *antecedent*) takes values in a set $\left\{1,\;\ldots ,\;v_{j}\right\}$ ($v_{j}>1$), and $c_{i}^{\Gamma }$ (called *consequent*) takes values in {1,…,w} (w>1). Now, let $x=\{x_{1},\;\ldots ,\;x_{p}\}$ be an observed example, with $x_{j}\in \left\{1,\;\ldots ,\;v_{j}\right\}$, for each j=1,…,p. We propose *maximum matching* as *reasoning method*, where the *compatibility degree* of the rule $R_{i}^{\Gamma }$ for the example x (denoted by $\varphi_{i}^{\Gamma }\left(x\right)$) is calculated as the number of attributes whose value coincides with that of the corresponding antecedent in $R_{i}^{\Gamma }$, that is

$$\varphi_{i}^{\Gamma }\left(x\right)=\sum_{j=1}^{p}\mu_{ij}^{\Gamma }\left(x\right)$$

where:

$$\mu_{ij}^{\Gamma }\left(x\right)=\left\{\begin{array}{cc} 1 & if\;x_{j}=b_{ij}^{\Gamma }\\ 0 & if\;x_{j}\neq b_{ij}^{\Gamma } \end{array}\right.$$

The *association degree* for the example x with a class c∈{1,…,w} is computed by adding the compatibility degrees for the example x of each rule $R_{i}^{\Gamma }$ whose consequent $c_{i}^{\Gamma }$ is equal to class *c*, that is:

$$\lambda_{c}^{\Gamma }\left(x\right)=\sum_{i=1}^{M}\eta_{ic}^{\Gamma }\left(x\right)$$

where:

$$\eta_{ic}^{\Gamma }\left(x\right)=\left\{\begin{array}{cc} \varphi_{i}^{\Gamma }\left(x\right) & if\;c=c_{i}^{\Gamma }\\ 0 & if\;c\neq c_{i}^{\Gamma } \end{array}\right.$$

Therefore, the *classification* (or output) of the classifier Γ for the example x corresponds to the class whose association degree is maximum, that is:

$$f^{\Gamma }\left(x\right)=\arg_{c}\max_{c=1}^{w}\lambda_{c}^{\Gamma }\left(x\right)$$

### 3.2. A Multi-Objective Optimization Solution

Let D be a dataset of *K* instances with *p* categorical input attributes, p>0, and a categorical output attribute. Each input attribute *j* can take a category $x_{j}\in \left\{1,\;\ldots ,\;v_{j}\right\}$, $v_{j}>1$, j=1,…,p, and the output attribute can take a class $c\in \left\{1,\;\ldots ,\;w\right\}$, w>1. The problem of finding an optimal classifier Γ, as described in the previous section, can be formulated as an instance of the multi-objective constrained problem in Equation (1) with two objectives and two constraints:

(3) $$\begin{array}{cc} Max./Min. & F_{D}\left(\Gamma \right)\\ Min. & \mathrm{NR}(\Gamma )\\ subject\;to: & \mathrm{NR}(\Gamma )\geq w\\ & \mathrm{NR}\left(\Gamma \right)\leq M_{max} \end{array}$$

**Figure 2:**
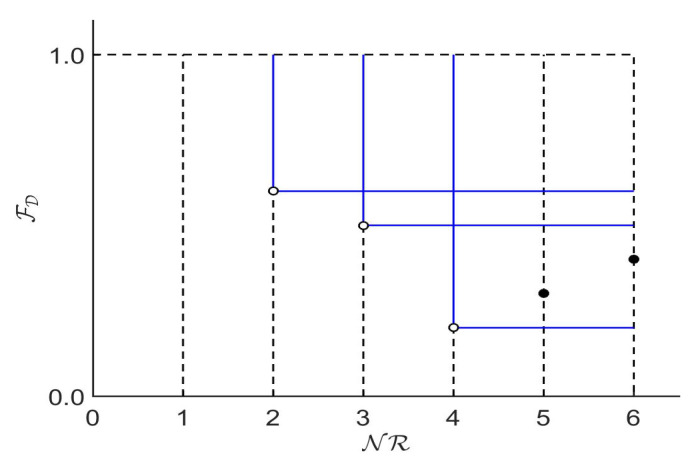
A Pareto front of a binary classification problem as formulated in Equation (3) where $F_{D}\left(\Gamma \right)$ is minimized and NR(Γ) is minimized.

In the problem (Equation (3)), the function $F_{D}\left(\Gamma \right)$ is a performance measure of the classifier Γ over the dataset D, the function NR(Γ) is the number of rules of the classifier Γ, and the constraints NR(Γ)≥w and $\mathrm{NR}\left(\Gamma \right)\leq M_{max}$ limit the number of rules of the classifier Γ to the interval $\left[w,M_{max}\right]$, where *w* is the number of classes of the output attribute and $M_{max}$ is given by a user. Objectives $F_{D}\left(\Gamma \right)$ and NR(Γ) are in conflict. The fewer rules the classifier has, the fewer instances it can cover, that is, if the classifier is simpler it will have less capacity for prediction. There is, therefore, an intrinsic conflict between problem objectives (e.g., maximize accuracy and minimize model complexity) which cannot be easily aggregated to a single objective. Both objectives are typically optimized simultaneously in many other classification systems, such as neural networks or decision trees [47,48]. Figure 2 shows the Pareto front of a dummy binary classification problem described as in Equation (3), with $M_{max}=6$ rules, where $F_{D}\left(\Gamma \right)$ is maximized. This front is composed of three non-dominated solutions (three possible classifiers) with two, three and four rules, respectively. The solutions with five and six rules are dominated (both by the solution with four rules).

Both *ENORA* and *NSGA-II* have been adapted to solve the problem described in Equation (3) with *variable-length representation* based on a *Pittsburgh approach*, *uniform random initialization*, *binary tournament selection*, *handling constraints*, ranking based on *non-domination level* with *crowding distance*, and *self-adaptive variation operators*. *Self-adaptive variation operators* work on different levels of the classifier: *rule crossover*, *rule incremental crossover*, *rule incremental mutation*, and *integer mutation*.

#### 3.2.1. Representation

We use a variable-length representation based on a Pittsburgh approach [49], where each individual *I* of a population contains a variable number of rules $M_{I}$, and each rule $R_{i}^{I}$, i=1,…, is codified in the following components:

- Integer values associated to the antecedents $b_{ij}^{I}\in \left\{1,\;\ldots ,\;v_{j}\right\}$, for $i=1,\;\ldots ,\;M_{I}$ and j=1,…,p.
- Integer values associated to the consequent $c_{i}^{I}\in \left\{1,\;\ldots ,\;w\right\}$, for $i=1,\;\ldots ,\;M_{I}$.

Additionally, to carry out self-adaptive crossing and mutation, each individual has two discrete parameters $d_{I}\in \left\{0,\;\ldots ,\;\delta \right\}$ and $e_{I}\in \left\{0,\;\ldots ,\;\epsilon \right\}$ associated with crossing and mutation, where δ≥0 is the number of crossing operators and ε≥0 is the number of mutation operators. Values $d_{I}$ and $e_{I}$ for self-adaptive variation are randomly generated from {0,δ} and {0,ϵ}, respectively. Table 1 summarizes the representation of an individual.

#### 3.2.2. Constraint Handling

The constraints NR(Γ)≥w and $\mathrm{NR}\left(\Gamma \right)\leq M_{max}$ are satisfied by means of specialized initialization and variation operators, which always generate individuals with a number of rules between *w* and $M_{max}$.

#### 3.2.3. Initial Population

The initial population (Algorithm 5) is randomly generated with the following conditions:

- Individuals are uniformly distributed with respect to the number of rules with values between *w* and $M_{max}$, and with an additional constraint that specifies that there must be at least one individual for each number of rules (Steps 4–8). This ensures an adequate initial diversity in the search space in terms of the second objective of the optimization model.
- All individuals contain at least one rule for any output class between 1 and *w* (Steps 16–20).

**Algorithm 5** Initialize population.**Require:**p>0 {Number of categorical input attributes} **Require:**
$v_{1},\;\ldots ,\;v_{p}$, $v_{j}>1$, j=1,…,p {Number of categories for the input attributes} **Require:**
w>1, {Number of classes for the output attribute} **Require:**
δ>0 {Number of crossing operators} **Require:**
ϵ>0 {Number of mutation operators} **Require:**
$M_{max}\geq w$ {Maximum number of rules} **Require:**
N>1 {Number of individuals in the population}
 1:P←∅ 2:**for**k=1 to *N* **do** 3: I← new Individual 4: **if** $k\leq M_{max}-w+1$ **then** 5:  $M_{I}\leftarrow k+w-1$ 6: **else** 7:  $M_{I}\leftarrow$ Int *Random*(*w*,$M_{max}$) 8: **end if** 9: {Random rule $R_{i}^{I}$}10: **for** i=1 to $M_{I}$ **do**11:  {Random integer values associated with the antecedents}12:  **for** j=1 to *p* **do**13:   $b_{ij}^{I}\leftarrow$*Random*(1,$v_{j}$)14:  **end for**15:  {Random integer value associated with the consequent}16:  **if** i<w **then**17:   $c_{i}^{I}=j$18:  **else**19:   $c_{i}^{I}\leftarrow$*Random*(1,*w*)20:  **end if**21: **end for**22: {Random integer values for adaptive variation}23: $d_{I}\leftarrow$*Random*(0,δ)24: $e_{I}\leftarrow$*Random*(0,ϵ)25: P←P∪I26:**end for**27:**return***P*


#### 3.2.4. Fitness Functions

Since the optimization model encompasses two objectives, each individual must be evaluated with two fitness functions, which correspond to the objective functions $F_{D}\left(\Gamma \right)$ and NR(Γ) of the problem (Equation (3)). The selection of the best individuals is done using the Pareto concept in a binary tournament.

#### 3.2.5. Variation Operators

We use *self-adaptive crossover and mutation*, which means that the selection of the operators is made by means of an adaptive technique. As we have explained (cf. Section 3.2.1), each individual *I* has two integer parameters $d_{I}\in \left\{0,\;\ldots ,\;\delta \right\}$ and $e_{I}\in \left\{0,\;\ldots ,\;\epsilon \right\}$ to indicate which crossover or mutation is carried out. In our case, δ=2 and ϵ=2 are two crossover operators and two mutation operators, so that $d_{I},e_{I}\in \left\{0,1,2\right\}$. Note that value 0 indicates that no crossover or no mutation is performed. Self-adaptive variation (Algorithm 6) generates two children from two parents by self-adaptive crossover (Algorithm 7) and self-adaptive mutation (Algorithm 8). Self-adaptive crossover of individuals I,J and self-adaptive mutation of individual *I* are similar to each other. First, with a probability $p_{v}$, the values $d_{I}$ and $e_{I}$ are replaced by a random value. Additionally, in the case of crossover, the value $d_{J}$ is replaced by $d_{I}$. Then, the crossover indicated by $d_{I}$ or the mutation indicated by $e_{I}$ is performed. In summary, if an individual comes from a given crossover or a given mutation, that specific crossover and mutation are preserved to their offspring with probability $p_{v}$, so the value of $p_{v}$ must be small enough to ensure a controlled evolution (in our case, we use $p_{v}=0.1$). Although the probability of the crossover and mutation is not explicitly represented, it can be computed as the ratio of the individuals for which crossover and mutation values are set to 1. As the population evolves, individuals with more successful types of crossover and mutation will be more common, so that the probability of selecting the more successful crossover and mutation types will increase. Using self-adaptive crossover and mutation operators helps to realize the goals of maintaining diversity in the population and sustaining the convergence capacity of the evolutionary algorithm, also eliminating the need of setting an a priori operator probability to each operator. In other approaches (e.g., [50]), the probabilities of crossover and mutation vary depending on the fitness value of the solutions.

Both *ENORA* and *NSGA-II* have been implemented with two crossover operators, *rule crossover* (Algorithm 9) and *rule incremental crossover* (Algorithm 10), and two mutation operators: *rule incremental mutation* (Algorithm 11) and *integer mutation* (Algorithm 12). *Rule crossover* randomly exchanges two rules selected from the parents, and *rule incremental crossover* adds to each parent a rule randomly selected from the other parent if its number of rules is less than the maximum number of rules. On the other hand, *rule incremental mutation* adds a new rule to the individual if the number of rules of the individual is less than the maximum number of rules, while *integer mutation* carries out a uniform mutation of a random antecedent belonging to a randomly selected rule.

**Algorithm 6** Variation.**Require:**Parent1, Parent2 {Individuals for variation}
 1:Child1←Parent1 2:Child1←Parent2 3:Self-adaptive crossover Child1, Child2 4:Self-adaptive mutation Child1 5:Self-adaptive mutation Child2 6:**return**Child1,Child2


**Algorithm 7** Self-adaptive crossover.**Require:***I*, *J* {Individuals for crossing} **Require:**
$p_{v}$ ($0<p_{v}<1$) {Probability of variation} **Require:**
δ>0 {Number of different crossover operators}
 1:**if** a random Bernoulli variable with probability $p_{v}$ takes the value 1 **then** 2: $d_{I}\leftarrow$*Random*(0,δ) 3:**end if** 4:$d_{J}\leftarrow d_{I}$ 5:Carry out the type of crossover specified by $d_{I}$:{0: No cross}{1: Rule crossover}{2: Rule incremental crossover}


**Algorithm 8** Self-adaptive mutation.**Require:***I* {Individual for mutation} **Require:**
$p_{v}$ ($0<p_{v}<1$) {Probability of variation} **Require:**
ϵ>0 {Number of different mutation operators}
 1:**if** a random Bernoulli variable with probability $p_{v}$ takes the value 1 **then** 2: $e_{I}\leftarrow$*Random*(0,ϵ) 3:**end if** 4:Carry out the type of mutation specified by $e_{I}$:{0: No mutation}{1: Rule incremental mutation}{2: Integer mutation}


**Algorithm 9** Rule crossover.**Require:***I*, *J* {Individuals for crossing}
 1:i←*Random*(1,$M_{I}$) 2:j←*Random*(1,$M_{J}$) 3:Exchange rules $R_{i}^{I}$ and $R_{j}^{J}$


**Algorithm 10** Rule incremental crossover.**Require:***I*, *J* {Individuals for crossing} **Require:**
$M_{max}$ {Maximum number of rules}
 1:**if**$M_{I}<M_{max}$**then** 2: j←*Random*(1,$M_{J}$) 3: Add $R_{j}^{J}$ to individual *I* 4:**end if** 5:**if**$M_{J}<M_{max}$**then** 6: i←*Random*(1,$M_{I}$) 7: Add $R_{i}^{I}$ to individual *J* 8:**end if**


**Algorithm 11** Rule incremental mutation.**Require:***I* {Individual for mutation} **Require:**
$M_{max}$ {Maximum number of rules}
 1:**if**$M_{I}<M_{max}$**then** 2: Add a new random rule to *I* 3:**end if**


**Algorithm 12** Integer mutation.**Require:***I* {Individual for mutation} **Require:**
p>0 {Number of categorical input attributes} **Require:**
$v_{1},\;\ldots ,\;v_{p}$,  $v_{j}>1$,  j=1,…,p {Number of categories for the input attributes}
 1:i←*Random*(1,$M_{I}$) 2:j←*Random*(1,*p*) 3:$b_{ij}^{I}\leftarrow$*Random*(1,$v_{j}$)


## 4. Experiment and Results

To ensure the reproducibility of the experiments, we have used publicly available datasets. In particular, we have designed two sets of experiments, one using the *Breast Cancer* [51] dataset, and the other using the *Monk’s Problem 2* [52] dataset.

### 4.1. The Breast Cancer Dataset

*Breast Cancer* encompasses 286 instances. Each instance corresponds to a patient who suffered from breast cancer and uses nine attributes to describe each patient. The class to be predicted is binary and represents whether the patient has suffered a recurring cancer event. In this dataset, 85 instances are positive and 201 are negative. Table 2 summarizes the attributes of the dataset. Among all instances, nine present some missing values; in the pre-processing phase, these have been replaced by the mode of the corresponding attribute.

### 4.2. The Monk’s Problem 2 Dataset

In July 1991, the monks of *Corsendonk Priory* attended a summer course that was being held in their priory, namely the 2nd European Summer School on Machine Learning. After a week, the monks could not yet clearly identify the best *ML* algorithms, or which algorithms to avoid in which cases. For this reason, they decided to create the three so-called *Monk’s problems*, and used them to determine which *ML* algorithms were the best. These problems, rather simple and completely artificial, became later famous (because of their peculiar origin), and have been used as a comparison for many algorithms on several occasions. In particular, in [53], they have been used to test the performance of state-of-the-art (at that time) learning algorithms such as *AQ17-DCI*, *AQ17-HCI*, *AQ17-FCLS*, *AQ14-NT*, *AQ15-GA*, *Assistant Professional*, *mFOIL*, *ID5R*, *IDL*, *ID5R-hat*, *TDIDT*, *ID3*, *AQR*, *CN2*, *WEB CLASS*, *ECOBWEB*, *PRISM*, *Backpropagation*, and *Cascade Correlation*. For our research, we have used the *Monk’s Problem 2*, which contains six categorical input attributes and a binary output attribute, summarized in Table 3. The target concept associated with the *Monk’s Problem 2* is the binary outcome of the logical formula:

*Exactly two of:* {heap_shape= round, body_shape=round, is_smiling=yes, holding=sword, jacket_color=red, has_tie=yes}

In this dataset, the original training and testing sets were merged to allow other sampling procedures. The set contains a total of 601 instances, and no missing values.

### 4.3. Optimization Models

We have conducted different experiments with different optimization models to calculate the overall performance of our proposed technique and to see the effect of optimizing different objectives for the same problem. First, we have designed a multi-objective constrained optimization model based on the *accuracy*:

(4) $$\begin{array}{cc} Max. & {\mathrm{ACC}}_{D}\left(\Gamma \right)\\ Min. & \mathrm{NR}(\Gamma )\\ subject\;to: & \mathrm{NR}(\Gamma )\geq w\\ & \mathrm{NR}\left(\Gamma \right)\leq M_{max} \end{array}$$

where ${\mathrm{ACC}}_{D}\left(\Gamma \right)$ is the proportion of correctly classified instances (both true positives and true negatives) among the total number of instances [54] obtained with the classifier Γ for the dataset D. ${\mathrm{ACC}}_{D}\left(\Gamma \right)$ is defined as:

$${\mathrm{ACC}}_{D}\left(\Gamma \right)=\frac{1}{K}\sum_{i=1}^{K}T_{D}\left(\Gamma ,i\right)$$

where *K* is the number of instances of the dataset D, and $T_{D}\left(\Gamma ,i\right)$ is the result of the classification of the instance *i* in D with the classifier Γ, that is:

$$T_{D}\left(\Gamma ,i\right)=\left\{\begin{array}{cc} 1 & if\;{\hat{c}}_{i}^{\Gamma }=c_{D}^{i}\\ 0 & if\;{\hat{c}}_{i}^{\Gamma }\neq c_{D}^{i} \end{array}\right.$$

where ${\hat{c}}_{i}^{\Gamma }$ is the predicted value of the *i*th instance in Γ, and $c_{D}^{i}$ is the corresponding true value in D. Our second optimization model is based on the *area under the ROC curve*:

(5) $$\begin{array}{cc} Max. & {\mathrm{AUC}}_{D}\left(\Gamma \right)\\ Min. & \mathrm{NR}(\Gamma )\\ subject\;to: & \mathrm{NR}(\Gamma )\geq w\\ & \mathrm{NR}\left(\Gamma \right)\leq M_{max} \end{array}$$

where ${\mathrm{AUC}}_{D}\left(\Gamma \right)$ is the area under the *ROC* curve obtained with the classifier Γ with the dataset D. The *ROC* (*Receiver Operating Characteristic*) curve [55] is a graphical representation of the *sensitivity* versus the *specificity* index for a classifier varying the *discrimination threshold* value. Such a curve can be used to generate statistics that summarize the performance of a classifier, and it has been shown in [54] to be a simple, yet complete, empirical description of the decision threshold effect, indicating all possible combinations of the relative frequencies of the various kinds of correct and incorrect decisions. The area under the *ROC* curve can be computed as follows [56]:

$${\mathrm{AUC}}_{D}\left(\Gamma \right)=\int_{0}^{1}S_{D}\left(\Gamma ,E_{D}^{-1}\left(\Gamma ,v\right)\right)dv$$

where $S_{D}\left(\Gamma ,t\right)$ (*sensitivity*) is the proportion of positive instances classified as positive by the classifier Γ in D, $1-E_{D}\left(\Gamma ,t\right)$ (*specificity*) is the proportion of negative instances classified as negative by Γ in D, and *t* is the discrimination threshold. Finally, our third constrained optimization model is based on the *root mean square error* (*RMSE*):

(6) $$\begin{array}{cc} Max./Min. & {\mathrm{RMSE}}_{D}\left(\Gamma \right)\\ Min. & \mathrm{NR}(\Gamma )\\ subject\;to: & \mathrm{NR}(\Gamma )\geq w\\ & \mathrm{NR}\left(\Gamma \right)\leq M_{max} \end{array}$$

where ${\mathrm{RMSE}}_{D}\left(\Gamma \right)$ is defined as the square root of the *mean square error* obtained with a classifier Γ in the dataset D:

$${\mathrm{RMSE}}_{D}\left(\Gamma \right)=\frac{1}{K}\sqrt{\sum_{i=1}^{K}{\left({\hat{c}}_{i}^{\Gamma }-c_{D}^{i}\right)}^{2}}$$

where ${\hat{c}}_{i}^{\Gamma }$ is the predicted value of the *i*th instance for the classifier Γ, and $c_{D}^{i}$ is the corresponding output value in the database D. Accuracy, area under the *ROC* curve, and root mean square error are all well-accepted measures used to evaluate the performance of a classifier. Therefore, it is natural to use such measures as fitting functions. In this way, we can establish which one behaves better in the optimization phase, and we can compare the results with those in the literature.

### 4.4. Choosing the Best Pareto Front

To compare the performance of *ENORA* and *NSGA-II* as metaheuristics in this particular optimization task, we use the *hypervolume metric* [57,58]. The hypervolume measures, simultaneously, the diversity and the optimality of the non-dominated solutions. The main advantage of using hypervolume against other standard measures, such as the *error ratio*, the *generational distance*, the *maximum Pareto-optimal front error*, the *spread*, the *maximum spread*, or the *chi-square-like deviation*, is that it can be computed without an optimal population, which is not always known [15]. The hypervolume is defined as the volume of the search space dominated by a population *P*, and is formulated as:

(7) $$HV\left(P\right)=\overset{\left|Q\right|}{\underset{i=1}{⋃}}v_{i}$$

where Q⊆P is the set of non-dominated individuals of *P*, and $v_{i}$ is the volume of the individual *i*. Subsequently, the *hypervolume ratio* (*HVR*) is defined as the ratio of the volume of the non-dominated search space over the volume of the entire search space, and is formulated as follows:

(8) $$HVR\left(P\right)=1-\frac{H\left(P\right)}{VS}$$

where VS is the volume of the search space. Computing *HVR* requires reference points that identify the maximum and minimum values for each objective. For *RBC* optimization, as proposed in this work, the following minimum ($F_{D}^{lower}$, ${\mathrm{NR}}^{lower}$) and maximum ($F_{D}^{upper}$, ${\mathrm{NR}}^{upper}$) points, for each objective, are set in the multi-objective optimization models in Equations (4)–(6):

$$F_{D}^{lower}=0,F_{D}^{upper}=1,{\mathrm{NR}}^{lower}=w,{\mathrm{NR}}^{upper}=M_{max}$$

**Figure 3:**
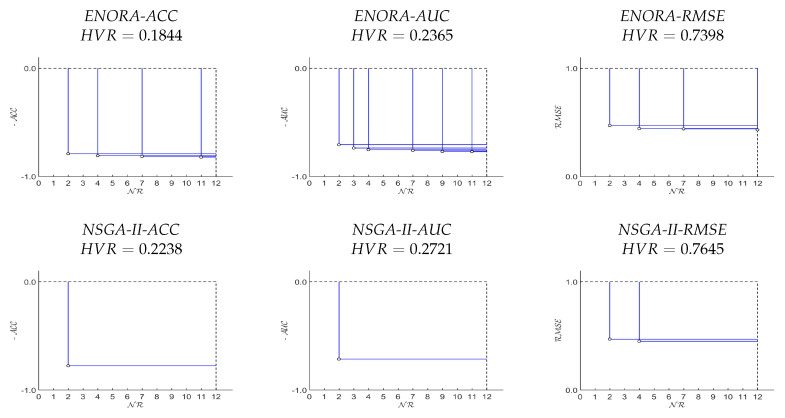
Pareto fronts of one execution of *ENORA* and *NSGA-II*, with $M_{max}=12$, on the *Breast Cancer* dataset, and their respective *HVR*. Note that in the case of multi-objective classification where $F_{D}$ is maximized (${\mathrm{ACC}}_{D}$ and ${\mathrm{AUC}}_{D}$), function $F_{D}$ has been converted to minimization for a better understanding of the Pareto front.

**Figure 4:**
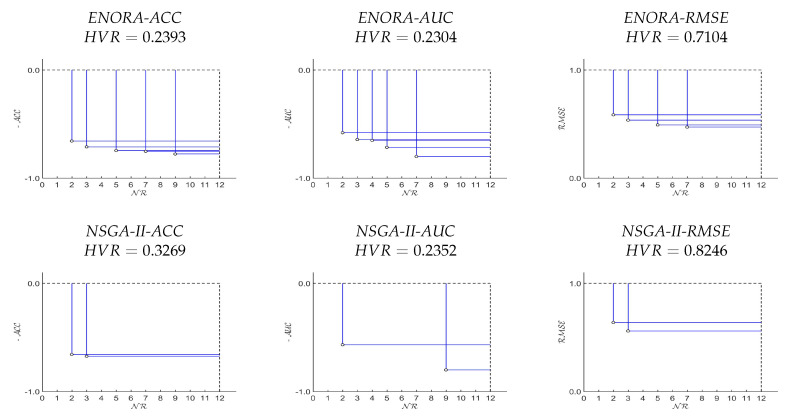
Pareto fronts of one execution of *ENORA* and *NSGA-II*, with $M_{max}=12$, on the *Monk’s Problem 2* dataset, and their respective *HVR*. Note that in the case of multi-objective classification where $F_{D}$ is maximized (${\mathrm{ACC}}_{D}$ and ${\mathrm{AUC}}_{D}$), function $F_{D}$ has been converted to minimization for a better understanding of the Pareto front.

A first single execution of all six models (three driven by *ENORA*, and three driven by *NSGA-II*), over both datasets, has been designed for the purpose of showing the aspect of the final Pareto front, and compare the hypervolume ratio of the models. The results of this single execution, with population size equal to 50 and 20,000 generations (1,000,000 evaluations in total), are shown in Figure 3 and Figure 4 (by default, $M_{max}$ is set to 10, to which we add 2, because both datasets have a binary class). Regarding the configuration of the number of generations and the size of the population, our criterion has been established as follows: once the number of evaluations is set to 1,000,000, we can decide to use a population size of 100 individuals and 10,000 generations, or to use a population size of 50 individuals and 20,000 generations. The first configuration (100 × 10,000) allows a greater diversity with respect to the number of rules of the classifiers, while the second one (50 × 20,000) allows a better adjustment of the classifier parameters and therefore, a greater precision. Given the fact that the maximum number of rules of the classifiers is not greater than 12, we think that 50 individuals are sufficient to represent four classifiers on average for each number of rules (4 × 12 = 48∼50). Thus, we prefer the second configuration (50× 20,000) because having more generations increases the chances of building classifiers with a higher precision.

Experiments were executed in a computer x64-based PC with one processor Intel64 Family 6 Model 60 Stepping 3 GenuineIntel 3201 Mhz, RAM 8131 MB. Table 4 shows the run time for each method over both datasets. Note that, although *ENORA* has less algorithmic complexity than *NSGA-II*, it has taken longer in experiments than *NSGA-II*. This is because the evaluation time of individuals in *ENORA* is higher than that of *NSGA-II* since *ENORA* has more diversity than *NSGA-II*, and therefore *ENORA* evaluates classifiers with more rules than *NSGA-II*.

From these results, we can deduce that, first, *ENORA* maintains a higher diversity of the population, and achieves a better hypervolume ratio with respect to *NSGA-II*, and, second, using accuracy as the first objective generates better fronts than using the area under the *ROC* curve, which, in turn, performs better than using the root mean square error.

### 4.5. Comparing Our Method with Other Classifier Learning Systems (Full Training Mode)

To perform an initial comparison between the performance of the classifiers obtained with the proposed method and the ones obtained with classical methods (*PART*, *JRip*, *OneR* and *ZeroR*), we have executed again the six models in full training mode.

The parameters have been configured as in the previous experiment (population size equal to 50 and 20,000 generations), excepting the $M_{max}$ parameter that was set to 2 for the *Breast Cancer* dataset (this case), while, for the *Monk’s Problem 2*, it was set to 9. Observe that, since $M_{min}=2$ in both cases, executing the optimization models using $M_{max}=2$ leads to a single objective search for the *Breast Cancer* dataset. In fact, after the preliminary experiments were run, it turned out that the classical classifier learning systems tend to return very small, although not very precise, set of rules on *Breast Cancer*, and that justifies our choice. On the other hand, executing the classical rule learners on *Monk’s Problem 2* returns more diverse sets of rules, which justifies choosing a higher $M_{max}$ in that case. To decide, a posteriori, which individual is chosen from the final front, we have used the default algorithm: the individual with the best value on the first objective is returned. In the case of *Monk’s Problem 2*, that individual has seven rules. The comparison is shown in Table 5 and Table 6, which show, for each classifier, the following information: *number of rules*, *percent correct*, *true positive rate*, *false positive rate*, *precision*, *recall*, *F-measure*, *Matthews correlation coefficient*, *area under the ROC curve*, *area under precision-recall curve*, and *root mean square error*. As for the *Breast Cancer* dataset (observe that the best result emerged from the proposed method), in the optimization model driven by *NSGA-II*, with root mean square error as the first objective (see Table 7), only *PART* was able to achieve similar results, although slightly worse, but at the price of having 15 rules, making the system clearly not interpretable. In the case of the *Monk’s Problem 2* dataset, *PART* returned a model with 47 rules, which is not interpretable by any standard, although it is very accurate. The best interpretable result is the one with seven rules returned by *ENORA*, driven by the root mean square error (see Table 8). The experiments for classical learners have been conducted using the default parameters.

### 4.6. Comparing Our Method with Other Classifier Learning Systems (Cross-Validation and Train/Test Percentage Split Mode)

To test the capabilities of our methodology in a more significant way, we proceeded as follows. First, we designed a *cross-validated* experiment for the *Breast Cancer* dataset, in which we iterated three times a 10-fold cross-validation learning process [59] and considered the average value of the performance metrics *percent correct*, *area under the ROC curve*, and *serialized model size* of all results. Second, we designed a *train/test percentage split* experiment for the *Monk’s Problem 2* dataset, in which we iterated ten times a 66% (training) versus 33% (testing) split and considered, again, the average result of the same metrics. Finally, we performed a statistical test over on results, to understand if they show any statistically significant difference. An execution of our methodology, and of standard classical learners, has been performed to obtain the models to be tested precisely under the same conditions of the experiment Section 4.5. It is worth observing that using two different types of evaluations allows us to make sure that our results are not influenced by the type of experiment. The results of the experiments are shown in Table 9 and Table 10.

The statistical tests aim to verify if there are significant differences among the means of each metric: *percent correct*, *area under the ROC curve* and *serialized model size*. We proceeded as follows. First, we checked normality and sphericity of each sample by means of the *Shapiro–Wilk normality test*. Then, if normality and sphericity conditions were met, we applied *one way repeated measures ANOVA*; otherwise, we applied the *Friedman test*. In the latter case, when statistically significant differences were detected, we applied the *Nemenyi post-hoc test* to locate where these differences were. Table A1, Table A2, Table A3, Table A4, Table A5, Table A6, Table A7, Table A8, Table A9, Table A10, Table A11 and Table A12 in Appendix A show the results of the performed tests for the *Breast Cancer* dataset for each of the three metrics, and Table A13, Table A14, Table A15, Table A16, Table A17, Table A18, Table A19, Table A20, Table A21, Table A22, Table A23 and Table A24 in Appendix B show the results for the *Monk’s Problem 2* dataset.

### 4.7. Additional Experiments

Finally, we show the results of the evaluation with 10-fold cross-validation for *Monk’s problem 2* dataset and for the following four other datasets:

1. *Tic-Tac-Toe-Endgame* dataset, with 9 input attributes, 958 instances, and binary class (Table 11).
2. *Car* dataset, with 6 input attributes, 1728 instances, and 4 output classes (Table 12).
3. *Chess (King-Rook vs. King-Pawn)* (*kr-vs-kp*), with 36 input attributes, 3196 instances, and binary class (Table 13).
4. *Nursery* dataset, with 8 input attributes, 12,960 instances, and 5 output classes (Table 14).

We have used the *ENORA* algorithm together with the ${\mathrm{ACC}}_{D}$ and ${\mathrm{RMSE}}_{D}$ objective functions in this case because these combinations have produced the best results for the *Breast Cancer* and *Monk’s problem 2* datasets evaluated in 10-fold cross-validation (population size equal to 50, 20,000 generations and $M_{max}=10\;+$ number of classes). Table 15 shows the results of the best combination *ENORA-ACC* or *ENORA-RMSE* together with the results of the classical rule-based classifiers.

## 5. Analysis of Results and Discussion

The results of our tests allow for several considerations. The first interesting observation is that *NSGA-II* identifies fewer solutions than *ENORA* on the Pareto front, which implies less diversity and therefore a worse hypervolume ratio, as shown in Figure 3 and Figure 4. This is not surprising: in several other occasions [19,34,60], it has been shown that *ENORA* maintains a higher diversity in the population than other well-known evolutionary algorithms, with generally positive influence on the final results. Comparing the results in full training mode against the results in cross-validation or in splitting mode makes it evident that our solution produces classification models that are more resilient to over-fitting. For example, the classifier learned by *PART* with *Monk’s Problem 2* presents a 94.01% accuracy in full training mode that drops to 73.51% in splitting mode. A similar, although with a more contained drop in accuracy, is shown by the classifier learned with *Breast Cancer* dataset; at the same time, the classifier learned by *ENORA* driven by accuracy shows only a 5.57% drop in one case, and even an improvement in the other case (see Table 5, Table 6, Table 9, and Table 10). This phenomenon is easily explained by looking at the number of rules: the more rules in a classifier, the higher the risk of over-fitting; *PART* produces very accurate classifiers, but at the price of adding many rules, which not only affects the interpretability of the model but also its resilience to over-fitting. Full training results seem to indicate that when the optimization model is driven by *RMSE* the classifiers are more accurate; nevertheless, they are also more prone to over-fitting, indicating that, on average, the optimization models driven by the accuracy are preferable.

From the statistical tests (whose results are shown in the Appendix A and Appendix B) we conclude that among the six variants of the proposed optimization model there are no statistical significative differences, which suggests that the advantages of our method do not depend directly on a specific evolutionary algorithm or on the specific performance measure that is used to drive the evolutions. Significant statistical differences between our method and very simple classical methods such as *OneR* were expectable. Significant statistical differences between our method and a well-consolidated one such as *PART* have not been found, but the price to be paid for using *PART* in order to have similar results to ours is a very high number of rules (15 vs. 2 in one case and 47 vs. 7 in the other case).

We would like to highlight that both the *Breast Cancer* dataset and the *Monk’s problem 2* dataset are difficult to approximate with interpretable classifiers and that none of the analyzed classifiers obtains high accuracy rates using the cross-validation technique. Even powerful black-box classifiers, such as *Random Forest* and *Logistic*, obtain success rates below 70% in 10-fold cross-validation for these datasets. However, *ENORA* obtains a better balance (trade-off) between precision and interpretability than the rest of the classifiers. For the rest of the analyzed datasets, the accuracy obtained using *ENORA* is substantially higher. For example, for the *Tic-Tac-Toe-Endgame* dataset, *ENORA* obtains a 98.3299% success percentage with only two rules in cross-validation, while *PART* obtains 94.2589% with 49 rules, and *JRip* obtains 97.8079% with nine rules. With respect to the results obtained in the datasets *Car*, *kr-vs-kp* and *Nursery*, we want to comment that better success percentage can be obtained if the maximum number of evaluations is increased. However, better success percentages imply a greater number of rules, which is to the detriment of the interpretability of the models.

## 6. Conclusions and Future Works

In this paper, we have proposed a novel technique for categorical classifier learning. Our proposal is based on defining the problem of learning a classifier as a multi-objective optimization problem, and solving it by suitably adapting an evolutionary algorithm to this task; our two objectives are minimizing the number of rules (for a better interpretability of the classifier) and maximizing a metric of performance. Depending on the particular metric that is chosen, (slightly) different optimization models arise. We have tested our proposal, in a first instance, on two different publicly available datasets, *Breast Cancer* (in which each instance represents a patient that has suffered from breast cancer and is described by nine attributes, and the class to be predicted represents the fact that the patient has suffered a recurring event) and *Monk’s Problem 2* (which is an artificial, well-known dataset in which the class to be predicted represents a logical function), using two different evolutionary algorithms, namely *ENORA* and *NSGA-II*, and three different choices as a performance metric, i.e., accuracy, the area under the *ROC* curve, and the root mean square error. Additionally, we have shown the results of the evaluation in 10-fold cross-validation of the publicly available *Tic-Tac-Toe-Endgame*, *Car*, *kr-vs-kp* and *Nursery* datasets.

Our initial motivation was to design a classifier learning system that produces interpretable, yet accurate, classifiers: since interpretability is a direct function of the number of rules, we conclude that such an objective has been achieved. As an aside, observe that our approach allows the user to decide, beforehand, a maximum number of rules; this can also be done in *PART* and *JRip*, but only indirectly. Finally, the idea underlying our approach is that multiple classifiers are explored at the same time in the same execution, and this allows us to choose the best compromise between the performance and the interpretability of a classifier a posteriori.

As a future work, we envisage that our methodology can benefit from an *embedded* future selection mechanism. In fact, all attributes are (ideally) used in every rule of a classifier learned by our optimization model. By simply relaxing such a constraint, and by suitably re-defining the first objective in the optimization model (e.g., by minimizing the sum of the lengths of all rules, or similar measures), the resulting classifiers will naturally present rules that use more features as well as rules that use less (clearly, the implementation must be adapted to obtain an initial population in which the classifiers have rules of different lengths as well as mutation operators that allow a rule to grow or to shrink). Although this approach does not follow the classical definition of feature selection mechanisms (in which a subset of features is selected that reduces the dataset over which a classifier is learned), it is natural to imagine that it may produce even more accurate classifiers, and more interpretable at the same time.

Currently, we are implementing our own version of *multi-objective differential evolution* (*MODE*) for rule-based classification for inclusion in the Weka Open Source Software issued under the GNU General Public License. The implementation of other algorithms, such as *MOEA/D*, their adaptation in the Weka development platform and subsequent analysis and comparison are planned for future work.

## Figures and Tables

**Table 1:** Chromosome coding for an individual *I*.

Codification for Rule Set					Codification for Adaptive Crossing and Mutation	
Antecedents				Consequent	Associated Crossing	Associated Mutation
$b_{11}^{I}$	$b_{21}^{I}$	…	$b_{q1}^{I}$	$c_{1}^{I}$		
⋮	⋮	⋮	⋮	⋮	$d_{I}$	$e_{I}$
$b_{1M_{I}}^{I}$	$b_{2M_{I}}^{I}$	…	$b_{qM_{I}}^{I}$	$c_{M_{I}}^{I}$		

**Table 2:** Attribute description of the *Breast Cancer* dataset.

#	Attribute Name	Type	Possible Values
1	age	categorical	10–19, 20–29, 30–39, 40–49, 50–59, 60–69, 70–79, 80–89, 90–99.
2	menopause	categorical	lt40, ge40, premeno
3	tumour-size	categorical	0–4, 5–9, 10–14, 15–19, 20–24, 25–29, 30–34, 35–39, 40–44, 45–49, 50–54, 55–59
4	inv-nodes	categorical	0–2, 3–5, 6–8, 9–11, 12–14, 15–17, 18–20, 21–23, 24–26, 27–29, 30–32, 33–35, 36–39
5	node-caps	categorical	yes, no
6	deg-malign	categorical	1, 2, 3
7	breast	categorical	left, right
8	breast-quad	categorical	left-up, left-low, right-up, right-low, central
9	irradiat	categorical	yes, no
10	class	categorical	no-recurrence-events, recurrence-events

**Table 3:** Attribute description of the *MONK’s Problem 2* dataset.

#	Atttribute Name	Type	Possible Values
1	head_shape	categorical	round, square, octagon
2	body_shape	categorical	round, square, octagon
3	is_smiling	categorical	yes, no
4	holding	categorical	sword, balloon, flag
5	jacket_color	categorical	red, yellow, green, blue
6	has_tie	categorical	yes, no
7	class	categorical	yes, no

**Table 4:** Run times of *ENORA* and *NSGA-II* for *Breast Cancer* and *Monk’s Problem 2* datasets.

Method	*Breast Cancer*	*Monk’s Problem 2*
*ENORA-ACC*	244.92 s.	428.14 s.
*ENORA-AUC*	294.75 s.	553.11 s.
*ENORA-RMSE*	243.30 s.	414.42 s.
*NSGA-II-ACC*	127.13 s.	260.83 s.
*NSGA-II-AUC*	197.07 s.	424.83 s.
*NSGA-II-RMSE*	134.87 s.	278.19 s.

**Table 5:** Comparison of the performance of the learning models in full training mode—*Breast Cancer* dataset.

Learning Model	Number of Rules	Percent Correct	*TP* Rate	*FP* Rate	Precision	Recall	*F*-Measure	*MCC*	*ROC* Area	*PRC* Area	*RMSE*
*ENORA-ACC*	2	79.02	0.790	0.449	0.796	0.790	0.762	0.455	0.671	0.697	0.458
*ENORA-AUC*	2	75.87	0.759	0.374	0.751	0.759	0.754	0.402	0.693	0.696	0.491
*ENORA-RMSE*	2	77.62	0.776	0.475	0.778	0.776	0.744	0.410	0.651	0.680	0.473
*NSGA-II-ACC*	2	77.97	0.780	0.501	0.805	0.780	0.738	0.429	0.640	0.679	0.469
*NSGA-II-AUC*	2	75.52	0.755	0.368	0.749	0.755	0.752	0.399	0.693	0.696	0.495
*NSGA-II-RMSE*	2	79.37	0.794	0.447	0.803	0.794	0.765	0.467	0.673	0.700	0.454
*PART*	15	78.32	0.783	0.397	0.773	0.783	0.769	0.442	0.777	0.793	0.398
*JRip*	3	76.92	0.769	0.471	0.762	0.769	0.740	0.389	0.650	0.680	0.421
*OneR*	1	72.72	0.727	0.563	0.703	0.727	0.680	0.241	0.582	0.629	0.522
*ZeroR*	-	70.27	0.703	0.703	0.494	0.703	0.580	0.000	0.500	0.582	0.457

**Table 6:** Comparison of the performance of the learning models in full training mode—*Monk’s Problem 2* dataset.

Learning Model	Number of Rules	Percent Correct	*TP* Rate	*FP* Rate	Precision	Recall	*F*-Measure	*MCC*	*ROC* Area	*PRC* Area	*RMSE*
*ENORA-ACC*	7	75.87	0.759	0.370	0.753	0.759	0.745	0.436	0.695	0.680	0.491
*ENORA-AUC*	7	68.71	0.687	0.163	0.836	0.687	0.687	0.523	0.762	0.729	0.559
*ENORA-RMSE*	7	77.70	0.777	0.360	0.777	0.777	0.762	0.481	0.708	0.695	0.472
*NSGA-II-ACC*	7	68.38	0.684	0.588	0.704	0.684	0.597	0.203	0.548	0.580	0.562
*NSGA-II-AUC*	7	66.38	0.664	0.175	0.830	0.664	0.661	0.497	0.744	0.715	0.580
*NSGA-II-RMSE*	7	68.71	0.687	0.591	0.737	0.687	0.595	0.226	0.548	0.583	0.559
*PART*	47	94.01	0.940	0.087	0.940	0.940	0.940	0.866	0.980	0.979	0.218
*JRip*	1	65.72	0.657	0.657	0.432	0.657	0.521	0.000	0.500	0.549	0.475
*OneR*	1	65.72	0.657	0.657	0.432	0.657	0.521	0.000	0.500	0.549	0.585
*ZeroR*	-	65.72	0.657	0.657	0.432	0.657	0.521	0.000	0.500	0.549	0.475

**Table 7:** Rule-based classifier obtained with *NSGA-II-RMSE* for *Breast Cancer* dataset.

Rule	Antecedents							Consequent
$R_{1}$:	*IF*	age = 50–59	*AND*	inv-nodes = 0–2	*AND*	node-caps = no		
	*AND*	deg-malig = 1	*AND*	breast = right	*AND*	breast-quad = left-low	*THEN*	class = no-recurrence-events
$R_{2}$:	*IF*	age = 60–69	*AND*	inv-nodes = 18–20	*AND*	node-caps = yes		
	*AND*	deg-malig = 3	*AND*	breast = left	*AND*	breast-quad = right-up	*THEN*	class = recurrence-events

**Table 8:** Rule-based classifier obtained with *ENORA-RMSE* for *Monk’s Problem 2* dataset.

Rule	Antecedents							Consequent
$R_{1}$:	*IF*	head_shape = round	*AND*	body_shape = round	*AND*	is_smiling = no		
	*AND*	holding = sword	*AND*	jacket_color = red	*AND*	has_tie = yes	*THEN*	class = yes
$R_{2}$:	*IF*	head_shape = octagon	*AND*	body_shape = round	*AND*	is_smiling = no		
	*AND*	holding = sword	*AND*	jacket_color = red	*AND*	has_tie = no	*THEN*	class = yes
$R_{3}$:	*IF*	head_shape = round	*AND*	body_shape = round	*AND*	is_smiling = no		
	*AND*	holding = sword	*AND*	jacket_color = yellow	*AND*	has_tie = yes	*THEN*	class = yes
$R_{4}$:	*IF*	head_shape = round	*AND*	body_shape = round	*AND*	is_smiling = no		
	*AND*	holding = sword	*AND*	jacket_color = red	*AND*	has_tie = no	*THEN*	class = yes
$R_{5}$:	*IF*	head_shape = square	*AND*	body_shape = square	*AND*	is_smiling = yes		
	*AND*	holding = flag	*AND*	jacket_color = yellow	*AND*	has_tie = no	*THEN*	class = no
$R_{6}$:	*IF*	head_shape = octagon	*AND*	body_shape = round	*AND*	is_smiling = yes		
	*AND*	holding = balloon	*AND*	jacket_color = blue	*AND*	has_tie = no	*THEN*	class = no
$R_{7}$:	*IF*	head_shape = octagon	*AND*	body_shape = octagon	*AND*	is_smiling = yes		
	*AND*	holding = sword	*AND*	jacket_color = green	*AND*	has_tie = no	*THEN*	class = no

**Table 9:** Comparison of the performance of the learning models in 10-fold cross-validation mode (three repetitions)—*Breast Cancer* dataset.

Learning Model	Percent Correct	*ROC* Area	Serialized Model Size
*ENORA-ACC*	73.45	0.61	9554.80
*ENORA-AUC*	70.16	0.62	9554.63
*ENORA-RMSE*	72.39	0.60	9557.77
*NSGA-II-ACC*	72.50	0.60	9556.20
*NSGA-II-AUC*	70.03	0.61	9555.70
*NSGA-II-RMSE*	73.34	0.60	9558.60
*PART*	68.92	0.61	55,298.13
*JRip*	71.82	0.61	7664.07
*OneR*	67.15	0.55	1524.00
*ZeroR*	70.30	0.50	915.00

**Table 10:** Comparison of the performance of the learning models in split mode—*Monk’s problem 2* dataset.

Learning Model	Percent Correct	*ROC* Area	Serialized Model Size
*ENORA-ACC*	76.69	0.70	9586.50
*ENORA-AUC*	72.82	0.79	9589.30
*ENORA-RMSE*	75.66	0.68	9585.30
*NSGA-II-ACC*	70.07	0.59	9590.60
*NSGA-II-AUC*	67.08	0.70	9619.70
*NSGA-II-RMSE*	67.63	0.54	9565.90
*PART*	73.51	0.79	73,115.90
*JRip*	64.05	0.50	5956.90
*OneR*	65.72	0.50	1313.00
*ZeroR*	65.72	0.50	888.00

**Table 11:** Attribute description of the *Tic-Tac-Toe-Endgame* dataset.

#	Attribute Name	Type	Possible Values
1	top-left-square	categorical	x, o, b
2	top-middle-square	categorical	x, o, b
3	top-right-square	categorical	x, o, b
4	middle-left-square	categorical	x, o, b
5	middle-middle-square	categorical	x, o, b
6	middle-right-square	categorical	x, o, b
7	bottom-left-square	categorical	x, o, b
8	bottom-middle-square	categorical	x, o, b
9	bottom-right-square	categorical	x, o, b
10	class	categorical	positive, negative

**Table 12:** Attribute description of the *Car* dataset.

#	Attribute Name	Type	Possible Values
1	buying	categorical	vhigh, high, med, low
2	maint	categorical	vhigh, high, med, low
3	doors	categorical	2, 3, 4, 5-more
4	persons	categorical	2, 4, more
5	lug_boot	categorical	small, med, big
6	safety	categorical	low, med, high
7	class	categorical	unacc, acc, good, vgood

**Table 13:** Attribute description of the *kr-vs-kp* dataset.

#	Attribute Name	Type	Possible Values
1	bkblk	categorical	t, f
2	bknwy	categorical	t, f
3	bkon8	categorical	t, f
4	bkona	categorical	t, f
5	bkspr	categorical	t, f
6	bkxbq	categorical	t, f
7	bkxcr	categorical	t, f
8	bkxwp	categorical	t, f
9	blxwp	categorical	t, f
10	bxqsq	categorical	t, f
11	cntxt	categorical	t, f
12	dsopp	categorical	t, f
13	dwipd	categorical	g, l
14	hdchk	categorical	t, f
15	katri	categorical	b, n, w
16	mulch	categorical	t, f
17	qxmsq	categorical	t, f
18	r2ar8	categorical	t, f
19	reskd	categorical	t, f
20	reskr	categorical	t, f
21	rimmx	categorical	t, f
22	rkxwp	categorical	t, f
23	rxmsq	categorical	t, f
24	simpl	categorical	t, f
25	skach	categorical	t, f
26	skewr	categorical	t, f
27	skrxp	categorical	t, f
28	spcop	categorical	t, f
29	stlmt	categorical	t, f
30	thrsk	categorical	t, f
31	wkcti	categorical	t, f
32	wkna8	categorical	t, f
33	wknck	categorical	t, f
34	wkovl	categorical	t, f
35	wkpos	categorical	t, f
36	wtoeg	categorical	n, t, f
37	class	categorical	won, nowin

**Table 14:** Attribute description of the *Nursery* dataset.

#	Attribute Name	Type	Possible Values
1	parents	categorical	usual, pretentious, great_pret
2	has_nurs	categorical	proper, less_proper, improper, critical, very_crit
3	form	categorical	complete, completed, incomplete, foster
4	children	categorical	1, 2, 3, more
5	housing	categorical	convenient, less_conv, critical
6	finance	categorical	convenient, inconv
7	social	categorical	nonprob, slightly_prob, problematic
8	health	categorical	recommended, priority, not_recom
9	class	categorical	not_recom, recommend, very_recom, priority, spec_prior

**Table 15:** Comparison of the performance of the learning models in 10-fold cross-validation mode—*Monk’s Problem 2*, *Tic-Tac-Toe-Endgame*, *Car*, *kr-vs-kp* and *Nursery* datasets.

Learning Model	Number of Rules	Percent Correct	*TP* Rate	*FP* Rate	Precision	Recall	*F*-Measure	*MCC*	*ROC* Area	*PRC* Area	*RMSE*
***Monk’s problem 2***											
*ENORA-ACC*	7	77.70	0.777	0.360	0.777	0.777	0.762	0.481	0.708	0.695	0.472
*PART*	47	79.53	0.795	0.253	0.795	0.795	0.795	0.544	0.884	0.893	0.380
*JRip*	1	62.90	0.629	0.646	0.526	0.629	0.535	−0.034	0.478	0.537	0.482
*OneR*	1	65.72	0.657	0.657	0.432	0.657	0.521	0.000	0.500	0.549	0.586
*ZeroR*	-	65.72	0.657	0.657	0.432	0.657	0.521	0.000	0.491	0.545	0.457
***Tic-Tac-Toe-Endgame***											
*ENORA-ACC/RMSE*	2	98.33	0.983	0.031	0.984	0.983	0.983	0.963	0.976	0.973	0.129
*PART*	49	94.26	0.943	0.076	0.942	0.943	0.942	0.873	0.974	0.969	0.220
*JRip*	9	97.81	0.978	0.031	0.978	0.978	0.978	0.951	0.977	0.977	0.138
*OneR*	1	69.94	0.699	0.357	0.701	0.699	0.700	0.340	0.671	0.651	0.548
*ZeroR*	-	65.35	0.653	0.653	0.427	0.653	0.516	0.000	0.496	0.545	0.476
***Car***											
*ENORA-RMSE*	14	86.57	0.866	0.089	0.866	0.866	0.846	0.766	0.889	0.805	0.259
*PART*	68	95.78	0.958	0.016	0.959	0.958	0.958	0.929	0.990	0.979	0.1276
*JRip*	49	86.46	0.865	0.064	0.881	0.865	0.870	0.761	0.947	0.899	0.224
*OneR*	1	70.02	0.700	0.700	0.490	0.700	0.577	0.000	0.500	0.543	0.387
*ZeroR*	-	70.02	0.700	0.700	0.490	0.700	0.577	0.000	0.497	0.542	0.338
***kr-vs-kp***											
*ENORA-RMSE*	10	94.87	0.949	0.050	0.950	0.949	0.949	0.898	0.950	0.927	0.227
*PART*	23	99.06	0.991	0.010	0.991	0.991	0.991	0.981	0.997	0.996	0.088
*JRip*	16	99.19	0.992	0.008	0.992	0.992	0.992	0.984	0.995	0.993	0.088
*OneR*	1	66.46	0.665	0.350	0.675	0.665	0.655	0.334	0.657	0.607	0.579
*ZeroR*	-	52.22	0.522	0.522	0.273	0.522	0.358	0.000	0.499	0.500	0.500
***Nursery***											
*ENORA-ACC*	15	88.41	0.884	0.055	0.870	0.884	0.873	0.824	0.915	0.818	0.2153
*PART*	220	99.21	0.992	0.003	0.992	0.992	0.992	0.989	0.999	0.997	0.053
*JRip*	131	96.84	0.968	0.012	0.968	0.968	0.968	0.957	0.993	0.974	0.103
*OneR*	1	70.97	0.710	0.137	0.695	0.710	0.702	0.570	0.786	0.632	0.341
*ZeroR*	-	33.33	0.333	0.333	0.111	0.333	0.167	0.000	0.500	0.317	0.370

## Abbreviations

The following abbreviations are used in this manuscript:

*ML*	Machine learning
*ANN*	Artificial neural networks
*DLNN*	Deep learning neural networks
*CEO*	Chief executive officer
*SVM*	Support vector machines
*IBL*	Instance-based learning
*DT*	Decision trees
*RBC*	Rule-based classifiers
*ROC*	Receiver operating characteristic
*RMSE*	Root mean square error performance metric
*FL*	Fuzzy logic
*MOEA*	Multi-objective evolutionary algorithms
*NSGA-II*	Non-dominated sorting genetic algorithm, 2nd version
*ENORA*	Evolutionary non-dominated radial slots based algorithm
*PART*	Partial decision tree classifier
*JRip*	*RIPPER* classifier of *Weka*
*RIPPER*	Repeated incremental pruning to produce error reduction
*OneR*	One rule classifier
*ZeroR*	Zero rule classifier
*ENORA-ACC*	*ENORA* with objective function defined as accuracy
*ENORA-AUC*	*ENORA* with objective function defined as area under the *ROC* curve
*ENORA-RMSE*	*ENORA* with *RMSE* objective function
*NSGA-II-ACC*	*NSGA-II* with objective function defined as accuracy
*NSGA-II-AUC*	*NSGA-II* with objective function defined as area under the *ROC* curve
*NSGA-II-RMSE*	*NSGA-II* with *RMSE* objective function
*HVR*	Hypervolume ratio
*TP*	True positive
*FP*	False positive
*MCC*	Matthews correlation coefficient
*PRC*	Precision-recall curve

## Appendix A. Statistical Tests for *Breast Cancer* Dataset

**Table A1:** Shapiro–Wilk normality test *p*-values for *percent correct* metric—*Breast Cancer* dataset.

Algorithm	*p*-Value	Null Hypothesis
*ENORA-ACC*	0.5316	Not Rejected
*ENORA-AUC*	0.3035	Not Rejected
*ENORA-RMSE*	0.7609	Not Rejected
*NSGA-II-ACC*	0.1734	Not Rejected
*NSGA-II-AUC*	0.3802	Not Rejected
*NSGA-II-RMSE*	0.6013	Not Rejected
*PART*	0.0711	Not Rejected
*JRip*	0.5477	Not Rejected
*OneR*	0.316	Not Rejected
*ZeroR*	**3.818 × 10${}^{-06}$**	Rejected

**Table A2:** Friedman *p*-value for *percent correct* metric—*Breast Cancer* dataset.

	*p*-Value	Null Hypothesis
Friedman	**5.111 × 10${}^{-04}$**	Rejected

**Table A3:** Nemenyi post-hoc procedure for *percent correct* metric—*Breast Cancer* dataset.

	*ENORA-ACC*	*ENORA-AUC*	*ENORA-RMSE*	*NSGA-II-ACC*	*NSGA-II-AUC*	*NSGA-II-RMSE*	*PART*	*JRip*	*OneR*
***ENORA-AUC***	0.2597	-	-	-	-	-	-	-	-
***ENORA-RMSE***	0.9627	0.9627	-	-	-	-	-	-	-
***NSGA-II-ACC***	0.9981	0.8047	1.0000	-	-	-	-	-	-
***NSGA-II-AUC***	0.2951	1.0000	0.9735	0.8386	-	-	-	-	-
***NSGA-II-RMSE***	1.0000	0.2169	0.9436	0.9960	0.2486	-	-	-	-
***PART***	0.1790	1.0000	0.9186	0.6997	1.0000	0.1461	-	-	-
***JRip***	0.9909	0.8956	1.0000	1.0000	0.9186	0.9840	0.8164	-	-
***OneR***	**0.0004**	0.6414	**0.0451**	**0.0108**	0.5961	**0.0002**	0.7546	**0.0212**	-
***ZeroR***	0.2377	1.0000	0.9538	0.7803	1.0000	0.1973	1.0000	0.8783	0.6709

**Table A4:** Summary of statistically significant differences for *percent correct* metric—*Breast Cancer* dataset.

	*ENORA-ACC*	*ENORA-RMSE*	*NSGA-II-ACC*	*NSGA-II-RMSE*	*JRip*
***OneR***	*ENORA-ACC*	*ENORA-RMSE*	*NSGA-II-ACC*	*NSGA-II-RMSE*	*JRip*

**Table A5:** Shapiro–Wilk normality test *p*-values for *area under the ROC curve* metric—*Breast Cancer* dataset.

Algorithm	*p*-Value	Null Hypothesis
*ENORA-ACC*	0.6807	Not Rejected
*ENORA-AUC*	0.3171	Not Rejected
*ENORA-RMSE*	0.6125	Not Rejected
*NSGA-II-ACC*	0.0871	Not Rejected
*NSGA-II-AUC*	0.5478	Not Rejected
*NSGA-II-RMSE*	0.6008	Not Rejected
*PART*	0.6066	Not Rejected
*JRip*	0.2978	Not Rejected
*OneR*	0.4531	Not Rejected
*ZeroR*	**0.0000**	Rejected

**Table A6:** Friedman *p*-value for *area under the ROC curve* metric—*Breast Cancer* dataset.

	*p*-Value	Null Hypothesis
Friedman	**8.232 × 10${}^{-10}$**	Rejected

**Table A7:** Nemenyi post-hoc procedure for *area under the ROC curve* metric—*Breast Cancer* dataset.

	*ENORA-ACC*	*ENORA-AUC*	*ENORA-RMSE*	*NSGA-II-ACC*	*NSGA-II-AUC*	*NSGA-II-RMSE*	*PART*	*JRip*	*OneR*
***ENORA-AUC***	1.0000	-	-	-	-	-	-	-	-
***ENORA-RMSE***	0.9972	0.9990	-	-	-	-	-	-	-
***NSGA-II-ACC***	0.9999	1.0000	1.0000	-	-	-	-	-	-
***NSGA-II-AUC***	1.0000	1.0000	1.0000	1.0000	-	-	-	-	-
***NSGA-II-RMSE***	0.9990	0.9997	1.0000	1.0000	1.0000	-	-	-	-
***PART***	0.9999	1.0000	1.0000	1.0000	1.0000	1.0000	-	-	-
***JRip***	1.0000	1.0000	0.9992	1.0000	1.0000	0.9998	1.0000	-	-
***OneR***	**0.0041**	**0.0062**	0.0790	**0.0323**	**0.0281**	0.0582	**0.0345**	**0.0067**	-
***ZeroR***	**3.8 × 10${}^{-07}$**	**7.2 × 10${}^{-07}$**	**4.6 × 10${}^{-05}$**	**9.8 × 10${}^{-06}$**	**7.8 × 10${}^{-06}$**	**2.7 × 10${}^{-05}$**	**1.1 × 10${}^{-05}$**	**8.1 × 10${}^{-07}$**	0.6854

**Table A8:** Summary of statistically significant differences for *area under the ROC curve* metric—*Breast Cancer* dataset.

	*ENORA-ACC*	*ENORA-AUC*	*ENORA-RMSE*	*NSGA-II-ACC*	*NSGA-II-AUC*	*NSGA-II-RMSE*	*PART*	*JRip*
***OneR***	*ENORA-ACC*	*ENORA-AUC*	-	*NSGA-II-ACC*	*NSGA-II-AUC*	-	*PART*	*JRip*
***ZeroR***	*ENORA-ACC*	*ENORA-AUC*	*ENORA-RMSE*	*NSGA-II-ACC*	*NSGA-II-AUC*	*NSGA-II-RMSE*	*PART*	*JRip*

**Table A9:** Shapiro–Wilk normality test *p*-values for *serialized model size* metric—*Breast Cancer* dataset.

Algorithm	*p*-Value	Null Hypothesis
*ENORA-ACC*	**5.042 × 10${}^{-05}$**	Rejected
*ENORA-AUC*	**2.997 × 10${}^{-07}$**	Rejected
*ENORA-RMSE*	**4.762 × 10${}^{-04}$**	Rejected
*NSGA-II-ACC*	**4.88 × 10${}^{-06}$**	Rejected
*NSGA-II-AUC*	**2.339 × 10${}^{-07}$**	Rejected
*NSGA-II-RMSE*	**2.708 × 10${}^{-06}$**	Rejected
*PART*	0.3585	Not Rejected
*JRip*	**9.086 × 10${}^{-03}$**	Rejected
*OneR*	**1.007 × 10${}^{-07}$**	Rejected
*ZeroR*	**0.0000**	Rejected

**Table A10:** Friedman *p*-value for *serialized model size* metric—*Breast Cancer* dataset.

	*p*-Value	Null Hypothesis
Friedman	**2.2 × 10${}^{-16}$**	Rejected

**Table A11:** Nemenyi post-hoc procedure for *serialized model size* metric—*Breast Cancer* dataset.

	*ENORA-ACC*	*ENORA-AUC*	*ENORA-RMSE*	*NSGA-II-ACC*	*NSGA-II-AUC*	*NSGA-II-RMSE*	*PART*	*JRip*	*OneR*
***ENORA-AUC***	0.9998	-	-	-	-	-	-	-	-
***ENORA-RMSE***	**0.0053**	**0.0004**	-	-	-	-	-	-	-
***NSGA-II-ACC***	0.3871	0.0942	0.8872	-	-	-	-	-	-
***NSGA-II-AUC***	0.8872	0.4894	0.3871	0.9988	-	-	-	-	-
***NSGA-II-RMSE***	**4.1 × 10${}^{-05}$**	**1.3 × 10${}^{-06}$**	0.9860	0.2169	**0.0244**	-	-	-	-
***PART***	**4.7 × 10${}^{-09}$**	**5.6 × 10${}^{-11}$**	0.1973	**0.0013**	**3.3 × 10${}^{-05}$**	0.8689	-	-	-
***JRip***	0.2712	0.6997	**1.2 × 10${}^{-08}$**	**7.0 × 10${}^{-05}$**	**0.0025**	**6.3 × 10${}^{-12}$**	**6.9 × 10${}^{-14}$**	-	-
***OneR***	**0.0062**	0.0546	**1.5 × 10${}^{-12}$**	**5.5 × 10${}^{-08}$**	**5.5 × 10${}^{-06}$**	**8.3 × 10${}^{-14}$**	**8.3 × 10${}^{-14}$**	0.9584	-
***ZeroR***	**1.9 × 10${}^{-05}$**	**0.0004**	**7.3 × 10${}^{-14}$**	**8.6 × 10${}^{-12}$**	**2.3 × 10${}^{-09}$**	**8.5 × 10${}^{-14}$**	**<2 × 10${}^{-16}$**	0.2377	0.9584

**Table A12:** Summary of statistically significant differences for *serialized model size* metric—*Breast Cancer* dataset.

	*ENORA-ACC*	*ENORA-AUC*	*ENORA-RMSE*	*NSGA-II-ACC*	*NSGA-II-AUC*	*NSGA-II-RMSE*	*PART*
***ENORA-RMSE***	*ENORA-ACC*	*NSGA-II-AUC*	-	-	-	-	-
***NSGA-II-RMSE***	*ENORA-ACC*	*ENORA-AUC*	-	-	*NSGA-II-AUC*	-	-
***PART***	*ENORA-ACC*	*ENORA-AUC*	-	*NSGA-II-ACC*	*NSGA-II-AUC*	-	-
***JRip***	-	-	*JRip*	*JRip*	*JRip*	*JRip*	*JRip*
***OneR***	*OneR*	-	*OneR*	*OneR*	*OneR*	*OneR*	*OneR*
***ZeroR***	*ZeroR*	*ZeroR*	*ZeroR*	*ZeroR*	*ZeroR*	*ZeroR*	*ZeroR*

## Appendix B. Statistical Tests for *Monk’s Problem 2* Dataset

**Table A13:** Shapiro–Wilk normality test *p*-values for *percent correct* metric—*Monk’s Problem 2* dataset.

Algorithm	*p*-Value	Null Hypothesis
*ENORA-ACC*	0.6543	Not Rejected
*ENORA-AUC*	0.6842	Not Rejected
*ENORA-RMSE*	**0.0135**	Rejected
*NSGA-II-ACC*	0.979	Not Rejected
*NSGA-II-AUC*	0.382	Not Rejected
*NSGA-II-RMSE*	**0.0486**	Rejected
*PART*	0.5671	Not Rejected
*JRip*	**0.075**	Rejected
*OneR*	**4.672 × 10${}^{-06}$**	Rejected
*ZeroR*	**4.672 × 10${}^{-06}$**	Rejected

**Table A14:** Friedman *p*-value for *percent correct* metric—*Monk’s Problem 2* dataset.

	*p*-Value	Null Hypothesis
Frideman	**1.292 × 10${}^{-07}$**	Rejected

**Table A15:** Nemenyi post-hoc procedure for *percent correct* metric—*Monk’s Problem 2* dataset.

	*ENORA-ACC*	*ENORA-AUC*	*ENORA-RMSE*	*NSGA-II-ACC*	*NSGA-II-AUC*	*NSGA-II-RMSE*	*PART*	*JRip*	*OneR*
***ENORA-AUC***	0.8363	-	-	-	-	-	-	-	-
***ENORA-RMSE***	1.0000	0.9471	-	-	-	-	-	-	-
***NSGA-II-ACC***	0.1907	0.9902	0.3481	-	-	-	-	-	-
***NSGA-II-AUC***	**0.0126**	0.6294	**0.0342**	0.9958	-	-	-	-	-
***NSGA-II-RMSE***	**0.0126**	0.6294	**0.0342**	0.9958	1.0000	-	-	-	-
***PART***	0.8714	1.0000	0.9631	0.9841	0.5769	0.5769	-	-	-
***JRip***	**2.1 × 10${}^{-06}$**	**0.0048**	**1.0 × 10${}^{-05}$**	0.1341	0.6806	0.6806	**0.0036**	-	-
***OneR***	**0.0001**	0.0743	**0.0006**	0.6032	0.9875	0.9875	0.0601	0.9984	-
***ZeroR***	**0.0001**	0.0743	**0.0006**	0.6032	0.9875	0.9875	0.0601	0.9984	1.0000

**Table A16:** Summary of statistically significant differences for *percent correct* metric—*Monk’s Problem 2* dataset.

	*ENORA-ACC*	*ENORA-AUC*	*ENORA-RMSE*	*PART*
***NSGA-II-AUC***	*ENORA-ACC*	-	*ENORA-RMSE*	-
***NSGA-II-RMSE***	*ENORA-ACC*	-	*ENORA-RMSE*	-
***JRip***	*ENORA-ACC*	*ENORA-AUC*	*ENORA-RMSE*	*PART*
***OneR***	*ENORA-ACC*	-	*ENORA-RMSE*	-
***ZeroR***	*ENORA-ACC*	-	*ENORA-RMSE*	-

**Table A17:** Shapiro–Wilk normality test *p*-values for *area under the ROC curve* metric—*Monk’s Problem 2* dataset.

Algorithm	*p*-Value	Null Hypothesis
*ENORA-ACC*	0.4318	Not Rejected
*ENORA-AUC*	0.7044	Not Rejected
*ENORA-RMSE*	**0.0033**	Rejected
*NSGA-II-ACC*	0.3082	Not Rejected
*NSGA-II-AUC*	**0.0243**	Rejected
*NSGA-II-RMSE*	0.7802	Not Rejected
*PART*	0.1641	Not Rejected
*JRip*	0.3581	Not Rejected
*OneR*	**0.0000**	Rejected
*ZeroR*	**0.0000**	Rejected

**Table A18:** Friedman *p*-value for *area under the ROC curve* metric—*Monk’s Problem 2* dataset.

	*p*-Value	Null Hypothesis
Frideman	**1.051 × 10${}^{-08}$**	Rejected

**Table A19:** Nemenyi post-hoc procedure for *area under the ROC curve* metric—*Monk’s Problem 2* dataset.

	*ENORA-ACC*	*ENORA-AUC*	*ENORA-RMSE*	*NSGA-II-ACC*	*NSGA-II-AUC*	*NSGA-II-RMSE*	*PART*	*JRip*	*OneR*
***ENORA-AUC***	0.8363	-	-	-	-	-	-	-	-
***ENORA-RMSE***	1.0000	0.7054	-	-	-	-	-	-	-
***NSGA-II-ACC***	0.8870	0.0539	0.9556	-	-	-	-	-	-
***NSGA-II-AUC***	1.0000	0.8544	1.0000	0.8713	-	-	-	-	-
***NSGA-II-RMSE***	0.5504	**0.0084**	0.7054	0.9999	0.5239	-	-	-	-
***PART***	0.7054	1.0000	0.5504	**0.0269**	0.7295	**0.0036**	-	-	-
***JRip***	**0.0238**	**2.3 × 10${}^{-05}$**	**0.0482**	0.6806	**0.0211**	0.9471	**7.0 × 10${}^{-06}$**	-	-
***OneR***	**0.0084**	**4.7 × 10${}^{-06}$**	**0.0186**	0.4715	**0.0073**	0.8363	**1.4 × 10${}^{-06}$**	1.0000	-
***ZeroR***	**0.0084**	**4.7 × 10${}^{-06}$**	**0.0186**	0.4715	**0.0073**	0.8363	**1.4 × 10${}^{-06}$**	1.0000	1.0000

**Table A20:** Summary of statistically significant differences for *area under the ROC curve* metric—*Monk’s Problem 2* dataset.

	*ENORA-ACC*	*ENORA-AUC*	*ENORA-RMSE*	*NSGA-II-ACC*	*NSGA-II-AUC*	*PART*	
***NSGA-II-RMSE***	-	*ENORA-AUC*	-	-	-	-	-
***PART***	-	-	-	*PART*	-	*PART*	-
***JRip***	*ENORA-ACC*	*ENORA-AUC*	*ENORA-RMSE*	-	*NSGA-II-AUC*	-	*PART*
***OneR***	*ENORA-ACC*	*ENORA-AUC*	*ENORA-RMSE*	-	*NSGA-II-AUC*	-	*PART*
***ZeroR***	*ENORA-ACC*	*ENORA-AUC*	*ENORA-RMSE*	-	*NSGA-II-AUC*	-	*PART*

**Table A21:** Shapiro–Wilk normality test *p*-values for *serialized model size* metric—*Monk’s Problem 2* dataset.

Algorithm	*p*-Value	Null Hypothesis
*ENORA-ACC*	**4.08 × 10${}^{-05}$**	Rejected
*ENORA-AUC*	**0.0002**	Rejected
*ENORA-RMSE*	**0.0094**	Rejected
*NSGA-II-ACC*	**0.0192**	Rejected
*NSGA-II-AUC*	**0.0846**	Rejected
*NSGA-II-RMSE*	**0.0037**	Rejected
*PART*	0.9721	Not Rejected
*JRip*	**0.0068**	Rejected
*OneR*	**0.0000**	Rejected
*ZeroR*	**0.0000**	Rejected

**Table A22:** Friedman *p*-value for *serialized model size* metric—*Monk’s Problem 2* dataset.

	*p*-Value	Null Hypothesis
Frideman	**2.657 × 10${}^{-13}$**	Rejected

**Table A23:** Nemenyi post-hoc procedure for *serialized model size* metric—*Monk’s Problem 2* dataset.

	*ENORA-ACC*	*ENORA-AUC*	*ENORA-RMSE*	*NSGA-II-ACC*	*NSGA-II-AUC*	*NSGA-II-RMSE*	*PART*	*JRip*	*OneR*
***ENORA-AUC***	1.0000	-	-	-	-	-	-	-	-
***ENORA-RMSE***	1.0000	1.0000	-	-	-	-	-	-	-
***NSGA-II-ACC***	1.0000	1.0000	1.0000	-	-	-	-	-	-
***NSGA-II-AUC***	0.9925	0.9696	0.9984	0.9841	-	-	-	-	-
***NSGA-II-RMSE***	0.8870	0.9556	0.7966	0.9267	0.2622	-	-	-	-
***PART***	0.2824	0.1752	0.3957	0.2246	0.9015	**0.0027**	-	-	-
***JRip***	0.1752	0.2824	0.1110	0.2246	**0.0084**	0.9752	**1.0 × 10${}^{-05}$**	-	-
***OneR***	**0.0211**	**0.0431**	**0.0110**	**0.0304**	**0.0004**	0.6552	**1.5 × 10${}^{-07}$**	0.9993	-
***ZeroR***	**0.0012**	**0.0031**	**0.0006**	**0.0020**	**1.0 × 10${}^{-05}$**	0.1907	**1.3 × 10${}^{-09}$**	0.9015	0.9993

**Table A24:** Summary of statistically significant differences for *serialized model size* metric—*Monk’s Problem 2* dataset.

	*ENORA-ACC*	*ENORA-AUC*	*ENORA-RMSE*	*NSGA-II-ACC*	*NSGA-II-AUC*	*NSGA-II-RMSE*	*PART*
***PART***	-	-	-	-	-	*NSGA-II-RMSE*	-
***JRip***	-	-	-	-	*JRip*	-	*JRip*
***OneR***	*OneR*	*OneR*	*OneR*	*OneR*	*OneR*	-	*OneR*
***ZeroR***	*ZeroR*	*ZeroR*	*ZeroR*	*ZeroR*	*ZeroR*	-	*ZeroR*

## Appendix C. Nomenclature

**Table A25:** Nomenclature table (Part I).

Symbol	Definition
*Equation (1): Multi-objective constrained optimization*	
$x_{k}$	k-th decision variable
x	Set of decision variables
$f_{i}\left(x\right)$	i-th objective function
$g_{j}\left(x\right)$	j-th constraint
l>0	Number of objectives
m>0	Number of constraints
w>0	Number of decision variables
X	Domain for each each decision variable $x_{k}$
$X^{w}$	Domain for the set of decision variables
F	Set of all feasible solutions
S	Set of non-dominated solutions or Pareto optimal set
$D\left(x^{\prime },x\right)$	Pareto domination function
*Equation (2): Rule-based classification for categorical data*	
D	Dataset
$x_{i}$	ith categorical input attribute in the dataset D
x	Categorical input attributes in the dataset D
*y*	Categorical output attribute in the dataset D
$\left\{1,\ldots ,v_{i}\right\}$	Domain of i-th categorical input attribute in the dataset D
$\left\{1,\ldots ,w\right\}$	Domain of categorical output attribute in the dataset D
p≥0	Number of categorical input attributes in the dataset D
Γ	Rule-based classifier
$R_{i}^{\Gamma }$	ith rule of classifier Γ
$b_{ij}^{\Gamma }$	Category for jth categorical input attribute and ith rule of classifier Γ
$c_{i}^{\Gamma }$	Category for categorical output attribute and ith rule of classifier Γ
$\varphi_{i}^{\Gamma }\left(x\right)$	Compatibility degree of the ith rule of classifier Γ for the example x
$\mu_{ij}^{\Gamma }\left(x\right)$	Result of the ith rule of classifier Γ and jth categorical input attribute $x_{j}$
$\lambda_{c}^{\Gamma }\left(x\right)$	Association degree of classifier Γ for the example x with the class *c*
$\eta_{ic}^{\Gamma }\left(x\right)$	Result of of the ith rule of classifier Γ for the example x with the class *c*
$f_{\Gamma }\left(x\right)$	Classification or output of the classifier Γ for the example x
*Equation (3): Multi-objective constrained optimization problem for rule-based classification*	
$F_{D}\left(\Gamma \right)$	Performance objective function of the classifier Γ in the dataset D
NR(Γ)	Number of rules of the classifier Γ
$M_{max}$	Maximum number of rules allowed for classifiers
*Equations (4)–(6): Optimization models*	
${\mathrm{ACC}}_{D}\left(\Gamma \right)$	*Acurracy*: proportion of correctly classified instances with the classifier Γ in the dataset D
*K*	Number of instances in the dataset D
$T_{D}\left(\Gamma ,i\right)$	Result of the classification of the ith instance in the dataset D with the classifier Γ
${\hat{c}}_{i}^{\Gamma }$	Predicted value of the ith instance in the dataset D with the classifier Γ
$c_{D}^{i}$	Corresponding true value for the ith instance in the dataset D.
${\mathrm{AUC}}_{D}\left(\Gamma \right)$	Area under the *ROC* curve obtained with the classifier Γ in the dataset D.
$S_{D}\left(\Gamma ,t\right)$	*Sensitivity*: proportion of positive instances classified as positive with the classifier Γ in the dataset D
$1-E_{D}\left(\Gamma ,t\right)$	*Specificity*: proportion of negative instances classified as negative with the classifier Γ in the dataset D
*t*	Discrimination threshold
${\mathrm{RMSE}}_{D}\left(\Gamma \right)$	Square root of the *mean square error* obtained with the classifier Γ in the dataset D

**Table A26:** Nomenclature table (Part II).

*Equations (7) and (8): Hypervolume metric*	
*P*	Population
Q⊆P	Set of non-dominated individuals of *P*
$v_{i}$	Volume of the search space dominated by the individual *i*
HV(P)	Hypervolume: volume of the search space dominated by population *P*
H(P)	Volume of the search space non-dominated by population *P*
HVR(P)	Hypervolume ratio: ratio of H(P) over the volume of the entire search space
VS	Volume of the search space
$F_{D}^{lower}$	Minimum value for objective $F_{D}$
$F_{D}^{upper}$	Maximum value for objective $F_{D}$
${\mathrm{NR}}^{lower}$	Minimum value for objective NR
${\mathrm{NR}}^{upper}$	Maximum value for objective NR
